# Transglutaminase-Mediated Cross-Linking of Tropoelastin to Fibrillin Stabilises the Elastin Precursor Prior to Elastic Fibre Assembly

**DOI:** 10.1016/j.jmb.2020.08.023

**Published:** 2020-10-02

**Authors:** Michael P. Lockhart-Cairns, Helena Newandee, Jennifer Thomson, Anthony S. Weiss, Clair Baldock, Anna Tarakanova

**Affiliations:** 1 Wellcome Centre for Cell-Matrix Research, Division of Cell-Matrix Biology and Regenerative Medicine, School of Biological Sciences, Faculty of Biology, Medicine and Health, University of Manchester, Manchester Academic Health Science Centre, Manchester M13 9PT, UK; 2 Department of Biomedical Engineering, School of Engineering, University of Connecticut, Storrs-Mansfield, CT 06269-3139, USA; 3 School of Molecular Bioscience, Bosch Institute, Charles Perkins Centre, The University of Sydney, NSW 2006, Australia; 4 Department of Mechanical Engineering, School of Engineering, University of Connecticut, Storrs-Mansfield, CT 06269-3139, USA

**Keywords:** tropoelastin, elastin, fibrillin, tissue transglutaminase, elastic fibres, TE, tropoelastin, EGF, epidermal growth factor, TB, TGFβ binding-like, TG2, transglutaminase-2, AUC, analytical ultracentrifugation, SEC, size exclusion chromatography, MALS, multi-angle light scattering, SAXS, small-angle X-ray scattering, REMD, Replica Exchange molecular dynamics, RMSF, root mean square fluctuation

## Abstract

Elastic fibres are essential components of all mammalian elastic tissues such as blood vessels, lung and skin, and are critically important for the mechanical properties they endow. The main components of elastic fibres are elastin and fibrillin, where correct formation of elastic fibres requires a fibrillin microfibril scaffold for the deposition of elastin. It has been demonstrated previously that the interaction between fibrillin and tropoelastin, the elastin precursor, increases the rate of assembly of tropoelastin. Furthermore, tropoelastin and fibrillin can be cross-linked by transglutaminase-2, but the function of cross-linking on their elastic properties is yet to be elucidated. Here we show that transglutaminase cross-linking supports formation of a 1:1 stoichiometric fibrillin–tropoelastin complex. SAXS data show that the complex retains features of the individual proteins but is elongated supporting end-to-end assembly. Elastic network models were constructed to compare the dynamics of tropoelastin and fibrillin individually as well as in the cross-linked complex. Normal mode analysis was performed to determine the structures' most energetically favourable, biologically accessible motions which show that within the complex, tropoelastin is less mobile and this molecular stabilisation extends along the length of the tropoelastin molecule to regions remote from the cross-linking site. Together, these data suggest a long-range stabilising effect of cross-linking that occurs due to the covalent linkage of fibrillin to tropoelastin. This work provides insight into the interactions of tropoelastin and fibrillin and how cross-link formation stabilises the elastin precursor so it is primed for elastic fibre assembly.

## Highlights

• Elastic fibres, essential for mammalian tissue elasticity, contain elastin and fibrillin.
• Tissue transglutaminase cross-links fibrillin and tropoelastin to form a complex.
• The complex has an elongated structure that supports end-to-end assembly.
• Cross-linking restricts the molecular motions of tropoelastin and fibrillin.
• Stabilisation may provide an initiating molecular mechanism for elastic fibre assembly.

## Graphical abstract

**Figure:**
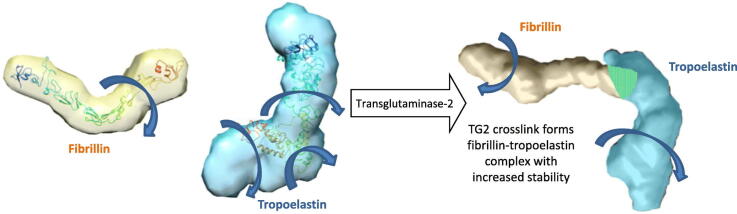

## Introduction

Elastic fibres are a major component of mammalian elastic tissues, providing resilience and flexibility to tissues such as arteries, the lungs and skin. These tissues can therefore expand and contract millions of times over the life-course, so they can carry out their essential elastic function. Elastic fibres have many components, but the two most abundant are elastin and fibrillin microfibrils [1]. Tropoelastin (TE), the 60-kDa soluble precursor to insoluble elastin, is characterised by alternating hydrophobic and hydrophilic domains and the TE monomer in solution has an extended, asymmetrical shape with a length of ~ 20 nm [2]. The N-terminal region is composed of an elongated “coil” where it eventually branches off to a “spur” region and bridges to a separate “foot” region containing the C terminus. The N-terminal “coil” region is primarily responsible for the elastic properties of TE, whilst the C-terminal region can interact with cell surface receptors [3]. TE has the capacity to spontaneously self-assemble or coacervate from a monomer to n-mer, which is reversible unless the assembled form is stabilised by lysyl oxidase cross-linking.

The other major component of elastic fibres is fibrillin microfibrils, whose secretion and assembly occurs independently from elastin [4]. Fibrillin microfibril deposition in developing elastic tissues precedes the expression of TE [1]. Fibrillin is secreted into the extracellular matrix, where it assembles to form 56-nm periodic beaded microfibrils [4]. Fibrillin is involved in the regulation of TGFβ and BMP family growth factors and can interact with integrins and syndecan cell surface receptors [[5], [6], [7]]. Whilst there are three distinct fibrillin genes in humans, fibrillin-1 is the most abundant and fibrillin-2 has an important role in development, whereas fibrillin-3 has a more restricted expression [[8], [9], [10]]. Mutations in fibrillin-1 cause Marfan syndrome, a disease characterised by severe skeletal, ocular and cardiovascular defects highlighting its essential role in elastic tissue function [11]. Fibrillin-1 is a large ~ 350 kDa glycoprotein composed of arrays of epidermal growth factor (EGF)-like domains interspersed with TGFβ binding-like (TB) domains [12]. Additionally, fibrillin-1 also contains a proline-rich region that has an as of yet undefined structure. Transglutaminase-2 (TG2) catalyses an enzymatic transamidation reaction that cross-links primary amines (e.g. lysine residues) to glutamine residues [13]. The interbead region of fibrillin microfibrils contains TG2 cross-links, which stabilise interactions between fibrillin molecules within the assembled microfibril [14]. Microfibrils are also associated with many binding proteins, including the microfibril-associated glycoproteins MAGP-1 and MAGP-2 (for a review, see Thomson *et al.* [1]). Specifically, MAGP-1 has been shown to bind to fibrillin-1, TE and other components in the extracellular matrix [[15], [16], [17]] and is also a substrate for TG2 [17].

The assembly of elastic fibres in the extracellular matrix is a highly organised and multifaceted process requiring the contribution of several matrix proteins. The amorphous core of insoluble elastic fibres comprises lysyl oxidase cross-linked TE deposited onto a scaffold of fibrillin microfibrils [18]. TE also directly interacts with the N-terminal region of fibrillin-1 via the TB2 domain and a covalent cross-link between TE and fibrillin can be formed by TG2 [19]. The cross-link between fibrillin and TE was mapped to a glutamine residue (Q669) in the TB2 domain of fibrillin and a lysine residue (K38) in domain 4 of TE, and addition of this region of fibrillin enhanced the coacervation of TE *in vitro* [20]. There is also a second high-affinity TE binding site in the central region of fibrillin-1 encompassing the TB3 domain [19]. The initial deposition of TE aggregates onto fibrillin microfibrils is believed to be the primary step required for further coacervation and an augmentation of TE globule recruitment to form larger elastic fibres. Once deposited on to fibrillin microfibrils, the TE aggregates coalesce to form stable, insoluble elastic fibres [18].

In order to understand the effect of TG2 cross-link formation on the structure and properties of TE and fibrillin, we have analysed the structural and biophysical properties of a fibrillin–TE complex. These data have also allowed us to explore the dynamics of TE and fibrillin individually and their interactions within the cross-linked assembly. Results show that specific movement patterns are found in the TE and fibrillin structures individually but are lost in the complex. This suggests a stabilising effect that occurs due to the cross-linking of fibrillin to TE and provides insight into the interactions of TE and fibrillin and the molecular mechanisms behind the process of elastic fibre assembly.

## Results

### Formation of a covalent fibrillin–tropoelastin complex cross-linked by TG2

**Figure 1:**
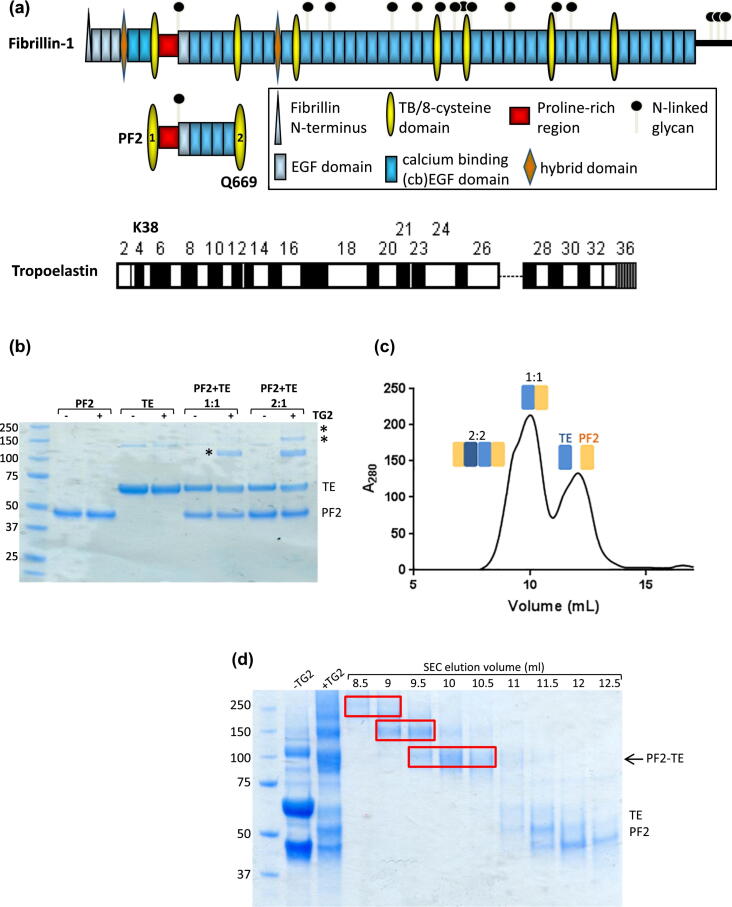
Formation of a covalent fibrillin–tropoelastin complex cross-linked by transglutaminase-2. (a) Schematic representation of the domain structures of fibrillin-1, the fibrillin-1 PF2 fragment used in this study and TE. The fibrillin domains are indicated with a key, and positions of N-linked glycans indicated based on sequence prediction or experimentally determined [21]. For TE, the domains are coloured white for hydrophobic and black for hydrophilic. The residues involved in the transglutaminase cross-link are Q669 in domain TB2 of fibrillin and K38 in domain 4 of TE [19,20]. (b) PF2 and TE were incubated alone, or at 1:1 or 2:1 ratio, in the presence (+) or absence (−) of TG2 (76.6 kDa). SDS-PAGE showing the formation of higher order species (*) after incubation of PF2 and TE in the presence of TG2, with a prominent species appearing at ~ 110 kDa. The input components, PF2 and TE, are labelled. (c) Following incubation of PF2 and TE with TG2 at a 1:1 ratio, the sample was separated by size exclusion chromatography (SEC) and the absorbance at 280 nm plotted by elution volume. Peaks corresponding to 2:2 PF2–TE and 1:1 PF2–TE species and uncomplexed TE and PF2 are indicated. (d) SDS-PAGE of the PF2–TE mixture before and after incubation with TG2, and SEC elution fractions shown in (c). SEC separates the higher order complexed species from the uncomplexed TE and PF2 components. The red boxes show the separated species that were labelled with asterisks in (b), including the ~ 110 kDa species that elutes at ~ 10 ml.

In order to determine the role of the TG2 cross-link between fibrillin and TE, a cross-linked complex was prepared for biophysical analysis. A fibrillin-1 construct encompassing domains TB1 to TB2 (Figure 1(a)), which includes residue Q669 involved in cross-link formation [20] was used for these studies (referred to as PF2 (molecular weight 44.2 kDa from protein sequence [22])). Full-length TE (molecular weight 60.0 kDa [23]) was also utilised, which contains residue K38 in domain 4, involved in cross-link formation with fibrillin. The cross-link formation between fibrillin PF2 and TE was optimised by screening cross-linking time, temperature and the ratios of PF2 and TE. At molar ratios where fibrillin to TE was greater than 1:1, higher-molecular-weight species were observed (Figure 1(b)). Whereas at a 1:1 ratio of PF2 to TE, a species of expected molecular weight for a 1:1 stoichiometric PF2–TE complex (~ 109 kDa including an N-linked glycan on PF2) was observed, which did not form when either PF2 or TE were incubated with TG2 individually. A trace amount of ~ 130–140 kDa species was present in the TE sample, in both the presence and the absence of TG2, possibly a trace amount of TE dimer. The addition of TG2 did not change the amount of this species so was not mediated by TG2. After cross-linking, the species were separated by size exclusion chromatography (SEC), which purified the cross-linked complex from uncomplexed TE and PF2 species and additional higher order species (Figure 1(c) and (d)).

The PF2 construct of fibrillin has an N-linked glycan at residue N448 that has been shown experimentally [21]. PF2 is expressed in HEK293-EBNA cells that add complex N-linked glycans with mass between 3 and 5 kDa [24]. To test whether the glycan was important for the interaction with TE or cross-linking, it was removed using PNGaseF digestion in native conditions, then the deglycosylated PF2 was subject to cross-linking with TE. In the presence of TG2, the same cross-linked species formed for both glycosylated and deglycosylated PF2, which indicated that the N-linked glycan on residue N448 of fibrillin did not play a role in cross-linking (Supplementary Figure 1). For all the following structural and hydrodynamic analysis, the natively glycosylated PF2 was utilised.

### Hydrodynamic analysis of the cross-linked 1:1 fibrillin–tropoelastin complex

**Figure 2:**
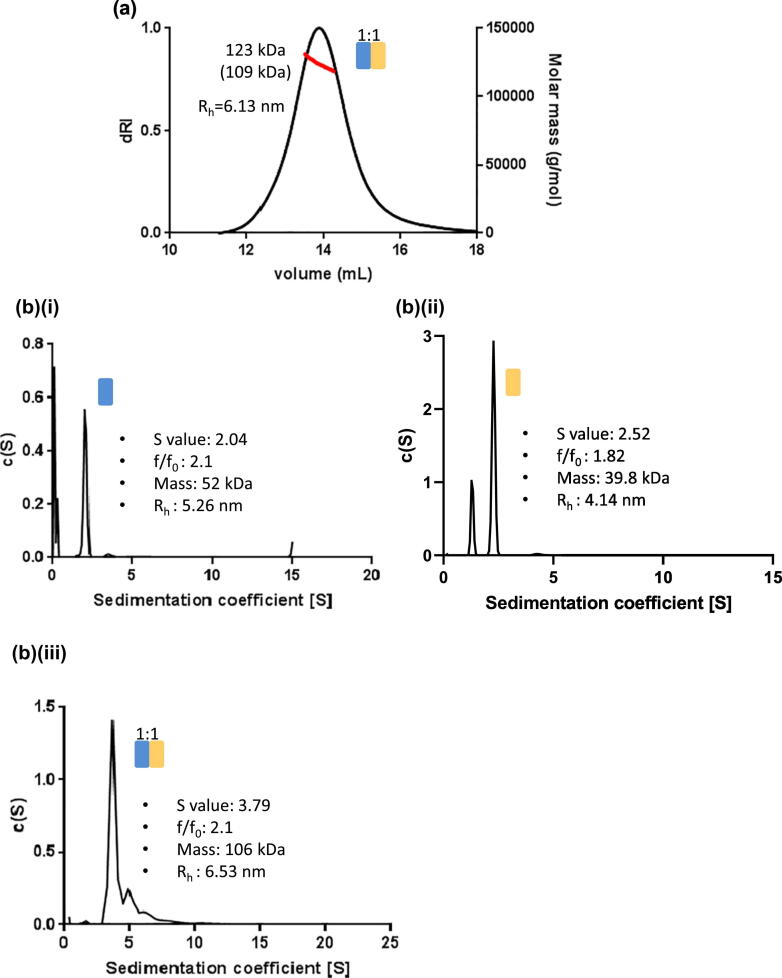
Hydrodynamic analysis of the cross-linked 1:1 fibrillin–tropoelastin complex. (a) Multi-angle light scattering analysis of the complex eluting at ~ 10 ml from SEC. Differential refractive index (dRI; black line) and molecular mass (red line) are shown as a function of elution volume after size exclusion using a Superose 6 column. Experimental MW is indicated, with theoretical MW of a 1:1 stoichiometric PF2–TE complex in brackets. (b) Sedimentation velocity analytical centrifugation (AUC) was used to determine the hydrodynamics of TE (i) and PF2 (ii) and compared to the complex (iii). The sedimentation coefficient (*S* value) for the complex (3.79) is larger than either TE (2.04) or PF2 alone (2.52), but the frictional ratio for TE and the complex is the same indicating that although the complex is larger there is a similar elongation factor.

To confirm whether a 1:1 TE–PF2 complex had formed, multi-angle light scattering (MALS) was performed to determine the mass of the complex. MALS showed that the complex had a hydrodynamic radius (*R_h_*) of 6.13 nm and molecular weight of 123 kDa, which is slightly larger than the expected mass of a 1:1 complex (~ 109 kDa with the addition of an N-linked glycan), but the experimentally determined mass can be influenced by the presence of trace amounts of larger species (Figure 2(a)). To gain further insight into the hydrodynamic properties of the TE–PF2 complex, sedimentation velocity analytical ultracentrifugation (AUC) was carried out on the complex and PF2 and TE alone (Figure 2(b)). As expected, the sedimentation coefficient (*S* value) for the TE–PF2 complex (3.79) is larger than TE (2.04) or PF2 (2.52) alone. Similarly, the *R_h_* of the TE–PF2 complex was 6.5 nm from AUC data (consistent with the value determined by MALS), whereas the *R_h_* values of TE and PF2 were smaller at 5.3 and 4.1 nm, respectively. However, the frictional ratio for TE and the TE–PF2 complex remains the same, which indicates that although the complex is larger, the complex and TE are both proportionately elongated, which would suggest that fibrillin and TE interact in an end-to-end manner.

### Structural analysis of the fibrillin–tropoelastin complex

**Figure 3:**
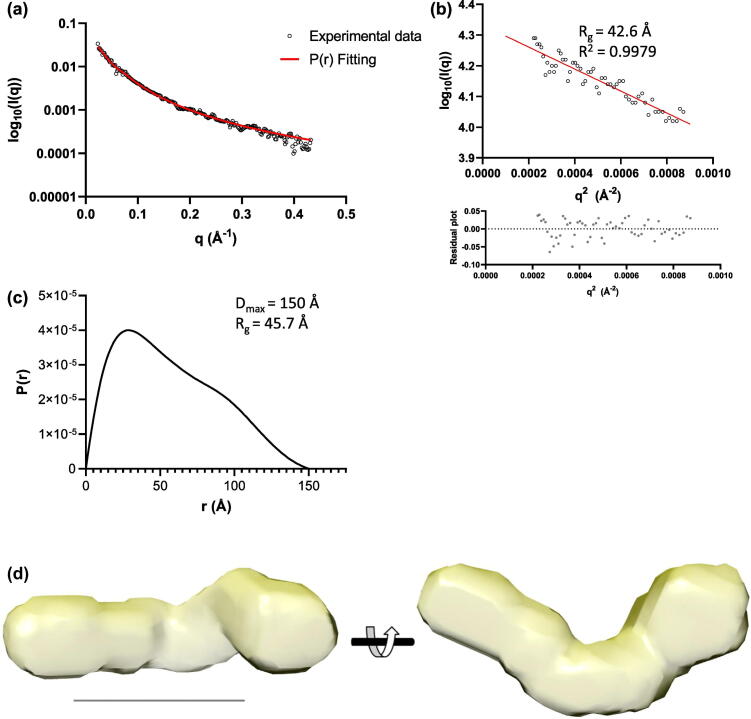
SAXS analysis of the fibrillin PF2 construct. (a) Experimental X-ray scattering data of the fibrillin PF2 construct plotted as a function of resolution. The indirect Fourier transform fitting to determine the pair-distance distribution function *P(r*) is shown in red. (b) The low *q* scattering data represented as a Guinier plot which is linear for values *q* ≤ 1/*R_g_*. From the Guinier plot, the radius of gyration (*R_g_*) can be estimated as 42.6 Å. (c) The pair-distance distribution function *P(r*) for the PF2 construct shows a maximum dimension of 150 Å. (d) Using DAMMIF, *ab initio* models of PF2 were calculated, the filtered average model is shown in two orthogonal orientations. The scale bar represents 10 nm.

**Figure 4:**
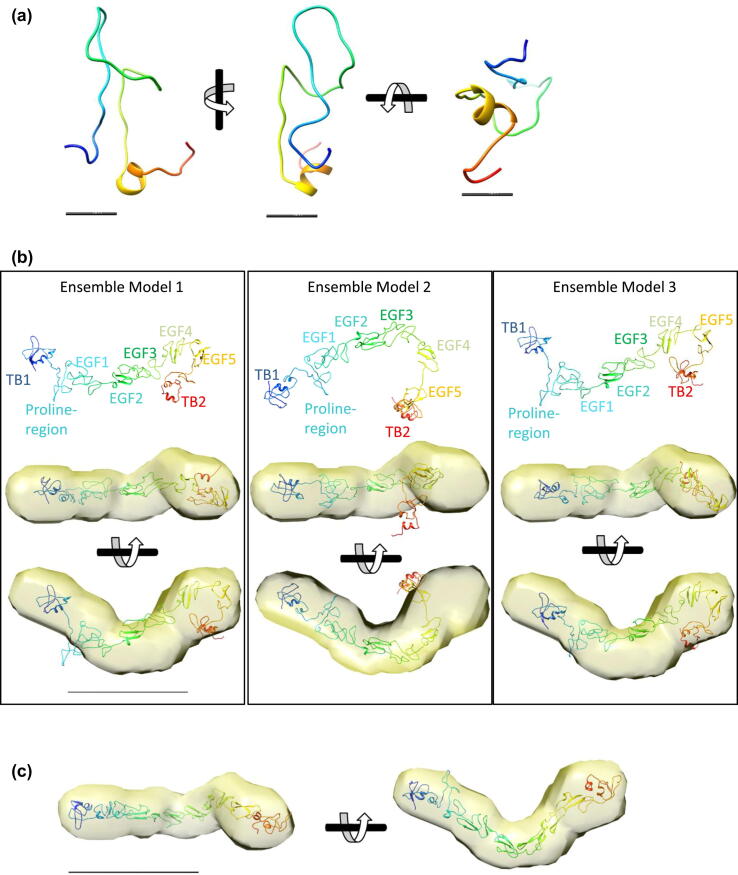
Structure of the proline-rich region and modelling of the PF2 construct. (a) Representative REMD structure of the 55-amino-acid proline-rich region of fibrillin-1. This representative structure is coloured from blue at the N terminus to red and the C terminus. The scale bar represents 10 Å. (b) Ensemble of three models of the PF2 region generated from rigid body modelling of the individual domains to the SAXS data using BilboMD. Each model was docked into the *ab initio* model (shown in yellow). The scale bar represents 10 nm. (c) Independent modelling of the fibrillin PF2 construct to the SAXS data. The model is coloured from blue at the N terminus (domain TB1) to red at the C terminus (domain TB2). The scale bar represents 10 nm.

Structural information on the complex was generated using small-angle X-ray scattering (SAXS). These data were collected on the TE–PF2 complex and each of the individual components. The SAXS data on the fibrillin PF2 construct and full-length TE were consistent with previously published measurements [2,25]. The SAXS data for PF2 was analysed and revealed a radius of gyration of 42.6 Å from the Guinier plot and the pair-distance distribution function (*P(r*)) showed a maximum dimension of 150 Å (Figure 3(a)–(c)). *Ab initio* modelling of PF2 showed an elongated curved shape with a widening at one end (Figure 3(d)). To model the domains within this envelope, homology models of the TB and EGF domains were generated. However, as no structural data are available for the proline-rich region, this 55-amino-acid region was modelled from an initial extended chain using Replica Exchange molecular dynamics (REMD) simulations. The proline-rich region of PF2 is likely to be flexible and may assume a variety of conformations, as indicated by the distribution of structures across clusters in the ensemble analysis (Supplementary Figure 2). The lowest-energy structure was extracted as a representative structure of the proline-rich region for modelling of the PF2 region. This region has dimensions of 3 nm × 1.5 nm × 1.5 nm (Figure 4(a)), little secondary structure with just a 5 amino acid section in an α-helical conformation and the remaining sequence adopting either β-turn or random coil conformation. Using the simulated structure of the proline-rich region and the TB and EGF homology models, the PF2 construct was modelled using the programme BilboMD [26]. An ensemble of three models was generated that represented the best fit to the experimental SAXS data (Supplementary Figure 3). The ensemble was composed of elongated structures that each had a curvature at the C-terminal end, around the second TB domain (Figure 4(b)). The individual models from the ensemble were docked into the PF2 *ab initio* density, and the models broadly fit the density; however, some regions of the density were unoccupied and two of the models had a hairpin-turn between domains cbEGF4–5 that would not be allowed sterically, given the short-linkers found between cbEGF domains. Therefore, the domains were manually docked into the *ab initio* density to provide a better representation of PF2 that was consistent with the SAXS envelope (Figure 4(c)). All models, in Figure 4(b) and (c), docked preferentially with the TB2 domain occupying the bulbous end of PF2, where the cross-link would occur.

**Figure 5:**
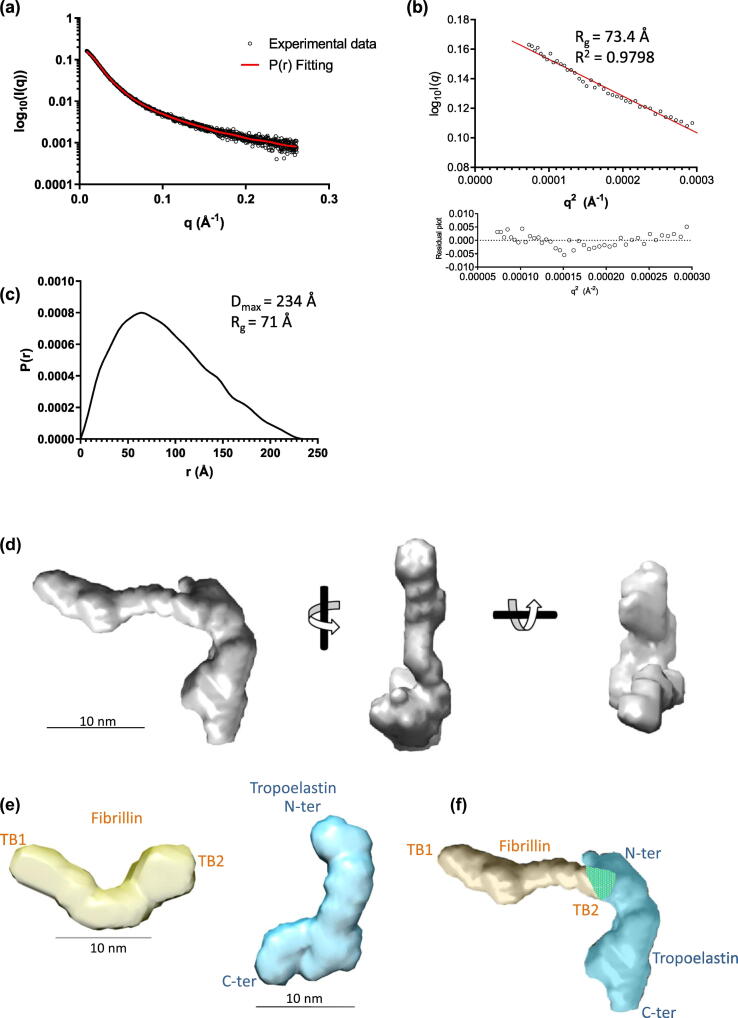
SAXS analysis of the fibrillin–tropoelastin complex. (a) Small-angle X-ray scattering data of the PF2–TE complex plotted as a function of resolution. The indirect Fourier transform fitting to determine the pair-distance distribution function *P(r*) is shown in red. (b) The low *q* scattering data represented as a Guinier plot which is linear for values *q* ≤ 1/*R_g_*. From the Guinier plot, the *R_g_* can be estimated as 73.4 Å. (c) The pair-distance distribution function *P(r*) for the PF2–TE complex shows a maximum dimension of 234 Å. (d) Using DAMMIF, *ab initio* models of the PF2–TE complex were calculated, the filtered average model is shown in three orthogonal orientations. The scale bar represents 10 nm. (e) The *ab initio* models of the PF2 region (yellow) and TE (blue) and (f) how they may be represented in the complex. The shape of which supports an interaction (shown in yellow/blue) between the N-terminal region of TE and the terminal domain of PF2.

The SAXS data for the complex were analysed and revealed a radius of gyration of 73.4 Å from the Guinier plot, and the pair-distance distribution function *P(r*) showed a maximum dimension of 234 Å (Figure 5(a)–(c)). *Ab initio* modelling of the complex was performed, and the resulting model was L-shaped with a longer, thin, elongated region connected perpendicularly to a shorter, wider region (Figure 5(d)). *Ab initio* modelling of TE revealed the characteristic shape with elongated N-terminal region and C-terminal foot (Supplementary Figure 4). Domain 4, where the cross-linking residue K38 is located, would reside in the elongated N-terminal part [27] (Figure 5(e)). Comparison of the *ab initio* models of the PF2 region, TE and the TE–PF2 complex, showed that the *ab initio* model of the complex had elements of both PF2 and TE combined and had dimensions consistent with the addition of these components (Figure 5(e)). Moreover, docking the N-terminal region of TE to the rounded end of PF2 gave a shape entirely consistent with the model of the complex which supports an interaction between the N-terminal region of TE and the terminal domain of PF2 (Figure 5(f)).

### Elastic network models for tropoelastin, PF2 and the TE–PF2 complex

**Figure 6:**
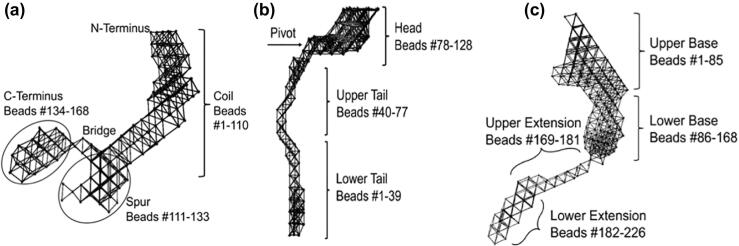
Elastic networks of tropoelastin, PF2 and the fibrillin–tropoelastin complex. Elastic network models, constructed based on small-angle X-ray scattering models for (a) TE, indicating the location of the N-terminal, coil, bridge, spur and C-terminal regions [2]; (b) PF2, indicating the location of the head, pivot, upper tail and lower tail regions; and (c) TE–PF2 complex, indicating the location of the upper base, lower base, upper extension and lower extension regions; Beads composing the structures are labelled numerically for reference.

To characterise the dynamic behaviour of TE, PF2 and the TE–PF2 complex, elastic network models were constructed based on SAXS models to allow the oscillating motions that describe normal modes of motion to be analysed. Beads that make up the elastic network model are distributed throughout the SAXS-derived models along a regular lattice. The beads are numbered along the structure's lattice so that bead mobility may be identifiably quantified. For TE, the elongated coil region consisting of the first 110 out of 168 beads, dominates the structure and displays varying types of motions. The spur, making up beads #111–133, is a continuation of the coil but the two separate regions are differentiated by a bridge. Protruding from the bridge is the C-terminal region, consisting of the last 35 beads (#134–168) of the structure. Together, the C-terminal and spur regions share coordinated bending movements with a pivot at the bridge [[27], [28], [29]] (Figure 6(a)).

The different regions of PF2 and TE–PF2 were labelled in a similar manner. The three main parts of the PF2 structure are the head, upper tail and lower tail (Figure 6(b)). The PF2 head can be distinguished from the tail because the 50 beads (#78–128) that make up the head form a globular clump that differs from the 77 beads that form the elongated tail. The head region often displays distinct movements from the upper and lower tail. For example, the PF2 head may be subjected to “twisting” dynamics about an imaginary axis that separates the head in half horizontally. The tail is longer and narrower than the head. The upper tail (beads #40–77) attaches to the head at bead #77. The lower tail is a continuation of the upper tail and makes up beads #1–39. A pivot of the bending motion separates the PF2 structure in two near bead #77 showing cooperative movement of the upper and lower tail regions.

The TE–PF2 complex has two distinct regions: the base and extension (Figure 6(c)). The large base, which consists of 168 beads of the total 226 beads, can be further separated into upper (beads #1–85) and lower (beads #86–168) base regions. The base tends to be very immobile across all modes. Trailing the base is the extension, which consists of 58 beads. The extension is also separated further into upper (beads #169–181) and lower (beads #182–226) extension regions. The two regions can be differentiated as the upper tail is narrower than the lower tail.

### Cross-linking to fibrillin imposes long-range stabilisation on tropoelastin

**Figure 7:**
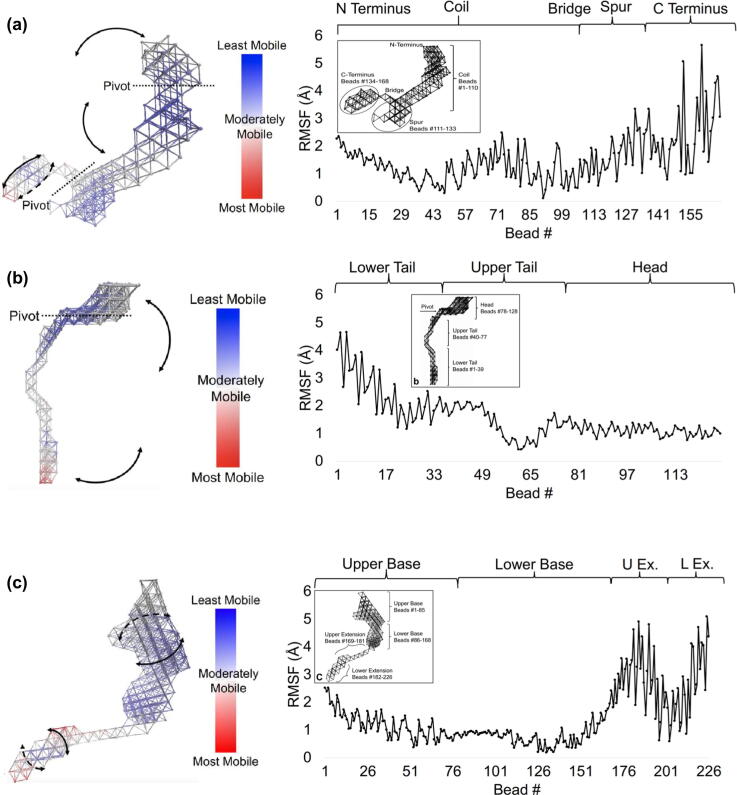
Cross-linking to fibrillin imposes long-range stabilisation on tropoelastin. Left panel: Normal mode analysis and root mean square fluctuation of beads of the elastic network model of combination of modes 1–6 of (a) TE, showing a bending coil; the dashed line and pivot indicates the axis about which bending occurs; (b) PF2, showing an overall bending motion; the dashed line and pivot indicates the axis about which bending occurs; and (c) TE–PF2, showing a twisting extension and twisting base; colours correspond to a mobility scale for low mobility (blue), moderate mobility (white) and high mobility (red). Right panel: Root mean square fluctuation of beads within elastic network models associated with combination of modes 1–6 of (a) TE (the inset shows the TE elastic network model showing its N terminus, coil, spur and C terminus along the TE structure), (b) PF2 (the inset shows the PF2 elastic network model showing head, upper tail and lower tail along the PF2 structure) and (c) TE–PF2 (the inset shows the TE–PF2 elastic network model showing upper base, lower base, upper extension and lower extension along the TE–PF2 structure).

The linear combination of the top six lowest-frequency normal modes was examined as representative of the molecules' native dynamics (Figure 7). To compare the mobility of different regions of the molecules, root mean square fluctuation (RMSF) is calculated per bead, numbered along the structure's regular lattice. As expected, TE exhibited a characteristic scissor-twist motion as previously described [[27], [28], [29], [30]]. The spur region and medial coil region display lower mobility as they are located proximally to a pivot, and regions of highest mobility within the TE structure are found at the N terminus (near bead #1) and C terminus (near bead #168), the most distal regions from the pivot. The maximum RMSF value is 5.658 Å, which is exhibited by bead #160 and located in the C-terminal region, whilst the minimum RMSF value is 0.132 Å, which is exhibited by bead #91 and located in the coil region (Figure 7(a)).

For PF2, dynamics were characterised by an overall bending motion perpendicular to its body with a pivot between the head and upper tail. The beads with the highest mobility are found at the terminal end of the structure (Figure 7(b)). The multiple twisting motions shown in TE do not transfer over to the TE–PF2 complex. The TE–PF2 complex, exhibits a stationary base and a mild twist in the lower extension, realised as a scrambling trend between local maxima and minima RMSF values between 0.186 and 2.518 Å for the base and between 0.596 and 5.086 Å for the extension (Figure 7(c)). The combination of TE and PF2 suggests a stabilising effect on the base of the complex, where the cross-linking site is expected to reside.

## Discussion

Here we describe the low-resolution structure, hydrodynamic properties and simulated dynamics of a transglutaminase cross-linked complex of TE and fibrillin. A number of elastic fibre proteins have been reported to be substrates of TG2 including fibrillin, TE and MAGP1 [14,17]. Fibrillin microfibrils contain transglutaminase cross-links that stabilise interactions between fibrillin molecules within the assembled microfibril [14]. Previously, we had shown that a transglutaminase cross-link between fibrillin and TE could be formed but the function of this cross-link was unknown [19]. Our data show that TE and the PF2 region of fibrillin forms a 1:1 complex when incubated with TG2. Higher ratios of fibrillin result in the formation of additional larger species. We did not include the region responsible for transglutaminase cross-linking in the fibrillin microfibril in our construct, so fibrillin–fibrillin cross-links did not occur.

In order to determine the structural consequences of the TG2 cross-link, a representative model for the PF2 construct was generated. Homology models were generated for the EGF and TB domains as structures of similar fibrillin domains have been determined [31,32]. The structure of the 55-amino-acid proline-rich region is unknown, this sequence has 43% proline content and thought to contain little secondary structure. Indeed, REMD simulations of this region resulted in a representative structure with little ordered secondary structure, with a 5-amino-acid section of α-helical conformation and the remaining sequence adopting either β-turn or random coil conformation. Modelling of the PF2 construct using the proline-rich region structure, predicted with REMD, and the other fibrillin domains was performed, which resulted in an elongated curved structure, reminiscent of earlier predictions performed without the proline-rich region structure [25].

AUC data on the PF2–TE complex show that the frictional ratio for TE and the complex remains the same, which indicates that they are both similarly elongated, which would suggest that fibrillin and TE interact in an end-to-end manner. Indeed, the SAXS data support this assembly showing an elongated L-shaped molecule which retained elements of the individual molecules' shapes. The SAXS models were used to generate elastic network models and perform normal mode analysis to simulate the dynamics of the individual components and the complex. Normal mode analysis was performed to extract the structures' least energetically expensive, biologically accessible motions. The oscillating motions that characterise each mode within TE, PF2 and the TE–PF2 complex were analysed for their dynamic patterns. Results show that specific movement patterns are found in the TE and PF2 structures individually but are not translated to the TE–PF2 complex.

**Figure 8:**
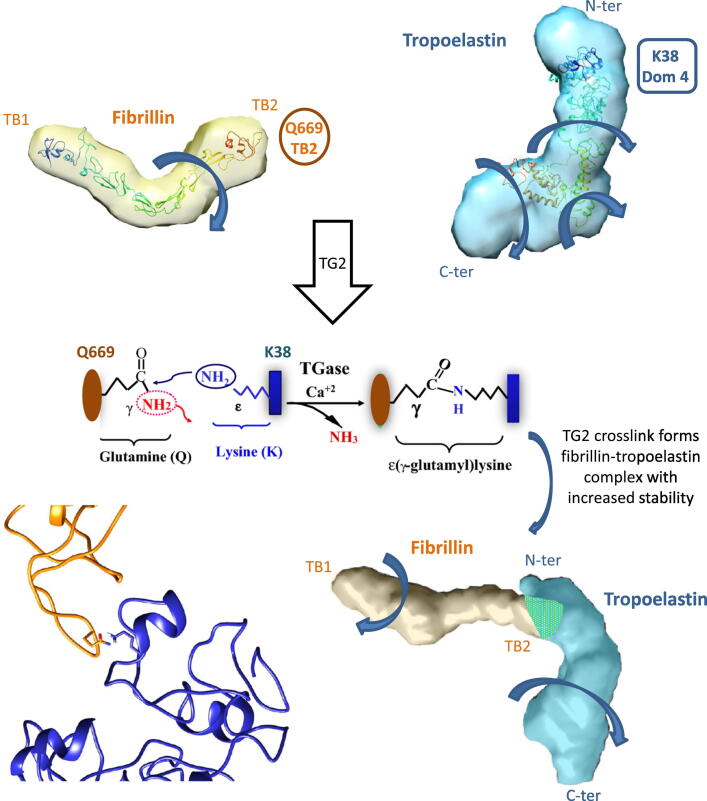
The multiple twisting motions seen in TE are not observed in the PF2–TE complex which is consistent with a model where the cross-link confers a stabilising effect on TE. Schematic diagram showing that residue Q669, in domain TB2 of fibrillin located in the head region of PF2, and residue K38, in domain 4 of TE, are covalently linked by TG2 in a transamidation reaction. These two residues are surface-exposed and accessible in the molecular models of the PF2 region (gold) and TE (blue) [27]. The multiple twisting motions seen in TE are not observed in the PF2–TE complex, which suggests that cross-link provides a stabilising effect to TE.

Within the complex, we propose TE as the head, and PF2 as the extension of TE–PF2 with the head of PF2 (the TB2 domain) affixed near the N terminus of TE. Evidence that supports this model is shown through the likeness of the motion between the tail of PF2 and the tail of TE–PF2 and the experimentally identified cross-link between TE and PF2 [19,20]. The consistent motions of PF2 and the tail of the TE–PF2 complex, suggest that TE is located in the head of the complex. These predictions are entirely consistent with the orientation of the PF2 models docked to the SAXS *ab initio* model in Figure 4 and our structural studies on TE. Indeed, in the molecular models of PF2 and TE, the residues involved in the cross-link are both surface exposed and can be brought into close proximity to allow a cross-link to form [2,27] (Figure 8). In modes 1–3, the head of TE–PF2 is immobile, and in modes 4–6, it displays limited fluctuation of the TE–PF2 base. This suggests a stabilising effect of the complex on TE. Stabilisation of TE by transglutaminase cross-linking to fibrillin may help to explain why in the presence of PF2, the temperature of coacervation is lowered and smaller changes in enthalpy and entropy of the coacervating system were observed [20].

The interaction between TE and PF2 was studied because of its role in the process of coacervation, the step in elastic fibre formation in which free, disordered TE monomers self-assemble to form aggregates [33]. This step helps to concentrate the monomers into large spherical globules and align TE in preparation for further cross-linking by lysyl oxidases. PF2 seems to improve the endothermic, entropy-driven process of TE coacervation as in the presence of PF2, the temperature of coacervation is decreased and the changes in enthalpy and entropy of the coacervating system were smaller [20]. TE can directly form a coacervate around PF2 rather than migrating to PF2, suggesting that PF2 is the site where TE is initially deposited on the microfibril and plays an early role in elastic fibre formation. This work provides insight into the interactions of TE and fibrillin and the initiating molecular mechanisms behind the process of elastic fibre assembly.

Fibrillin and tropoelastin are the dominant constituents of microfibrils and elastin, respectively. As the crosslink between fibrillin and tropoelastin restricts molecular movement, we propose that the microfibril–elastin interface similarly displays reduced motion. In this model, the stiffened interface helps to dissipate substantial amounts of elastic energy by structural damping [34]. The interface between tropoelastin and fibrillin may act as a structural damper to couple the mechanical flexibility required of elastin, and dissipative function. Furthermore, this stabilisation of tropoelastin by binding to fibrillin microfibrils likely contributes to the mechanical response of mature elastic fibres. In simulations of mechanical stretching of monomeric tropoelastin (unpublished), we observe a combination of twisting and stretching behaviour that contributes to tropoelastin's elasticity. We propose that tropoelastin–fibrillin binding contributes to the deformation mechanism observed in the mature fibre. This will be an interesting question to investigate in future studies.

## Methods

### Protein expression and purification

The fibrillin-1 PF2 fragment encoded by exons 9–17 was expressed in HEK293-EBNA cells as previously described [25]. PF2 was harvested from serum-free media and purified by nickel affinity chromatography followed by SEC on a Superdex 200 10/300 increase column in 10 mM Tris–HCl (pH 7.4), 150 mM NaCl. TE was expressed in bacteria and purified as described previously [23]. SHELΔ26A (Synthetic Human Elastin without domain 26A) corresponds to amino acid residues 27–724, the 60 kDa mature form of the secreted protein following removal of the signal peptide.

### Cross-link formation

To prepare the fibrillin–TE complex, samples were dialysed into 10 mM Hepes, 500 mM NaCl, 1 mM CaCl_2_ (pH 7.4), then 500 μg PF2 was incubated with 500 μg TE at 16 °C for 2 h. Following incubation, TG2 from guinea pig liver (Merck) was added with 1 mM CaCl_2_ and incubated for a further 4 h. The complex was then separated using SEC on a Superose 6 column in 10 mM Hepes, 500 mM NaCl, 1 mM CaCl_2_ (pH 7.4).

### MALS

Samples (0.5 mg/ml) were loaded onto a Superose 6 column, running at a flow rate of 0.75 ml/min, equilibrated with 10 mM Tris–HCl (pH 7.4), 150 mM NaCl. Eluted samples passed through a DAWN Wyatt Helios II 18-angle laser photometer with one of the detectors replaced with a Wyatt QELS detector coupled to a Wyatt Optilab rEX refractive index detector. The molecular mass and hydrodynamic radii of the resulting peaks were analysed using Astra 6.1 (Wyatt, Santa Barbara, USA).

### AUC

Sedimentation velocity AUC of PF2, TE and the PF2–TE complex was carried out in an XL-A Ultracentrifuge with an An50Ti-4-hole rotor (Beckman Coulter). Samples were measured at a wavelength of 230 nm at 45,000 rpm at 20 °C where sedimentation was scanned every 90 s for 200 scans. Data were analysed with Sedfit and Sednterp (http://sednterp.unh.edu/).

### SAXS

SAXS data on PF2 and TE collected on ID02 at the ESRF have already been published [2,25]. For this study, new SAXS data on PF2 and TE, as well as the PF2–TE complex, were also collected on beamline B21 at Diamond Light Source and compared to previously published data and models. The data collection parameters, sample concentrations and buffer conditions are detailed in Supplementary Table 1. At Diamond Light Source beamline B21, SAXS intensity data, *I*(*q*) *versus q* (*q* = 4*π*.  *sin* 2*θλ*) on TE, PF2 and the TE–PF2 complex, and matched buffers, were collected using the BioSAXS robot at 3-s intervals on a Pilatus 2M detector at a sample-to-detector distance of 4.0 m and wavelength of 1 Å. The scattering images obtained were spherically averaged using in-house software. For each sample, ten images were collected to check for radiation damage and protein aggregation between frames. Identical frames were merged and buffer scattering intensities subtracted using ScAtter. Particle shapes were generated *ab initio* using GASBOR (for TE) [35] or DAMMIF (for PF2 and the PF2–TE complex). Multiple *ab initio* runs were performed to generate 20 similar shapes that were combined and filtered to produce averaged models using the DAMAVER software package. The new SAXS data and models generated from these data for PF2 and TE were compared for similarity to the previously published data and models. Data on PF2, TE and the PF2–TE complex have been deposited in the SASDB (SAS2517, 2518, 2519).

### REMD of the proline-rich region

The 55-residue proline-rich sequence of fibrillin-1 (from Pro392 to Pro446) was simulated using REMD simulations [36]. All simulations were carried out using GROMACS version 5.1.2, with the CHARMM36m force field [37,38] and the TIP3P water model [39]. The LINCS algorithm [40] was used to constrain covalent bonds with hydrogen atoms. The short-range electrostatic interactions and Lennard–Jones interactions were evaluated with a cutoff of 10 Å. Particle-mesh Ewald summation [41] was used to calculate long range electrostatic interactions with a grid spacing of 1.6 Å and a fourth-order interpolation. The initial structure was built from a linear extended chain using with Avogadro [42]. The molecule was placed into a rectangular water box, 24 nm × 5 nm × 5 nm, with periodic boundary conditions. Chloride counterions were used in the simulation to neutralise the system. The structure was minimised through the steepest descent algorithm, followed by an initialization run for 1000 ps at 300 K in the canonical ensemble. Next, 160 temperature replicas were used, exponentially distributed in the temperature range 273 to 500 K. Exchanges were attempted every 0.5 ps to allow for system relaxation. Each replica was simulated for 50 ns, for a total simulation time of 8 μs, with a time step of 2 fs. Calculation of the root mean square deviation indicated convergence of the final structural ensemble structure from the initial extended chain. An ensemble of structures from the last 1000 exchanges at 300 K was analysed, corresponding to the experimental temperature. Clusters based on mutual similarity by root mean square deviation were created with the K-means clustering algorithm in the MMTSB tool set (Supplementary Figure 2) [43]. The lowest-energy structure from the most populated cluster was extracted as a representative structure.

### Ensemble modelling of PF2 to the SAXS data

The regions considered as rigid bodies were TB1 (330–390), proline-rich (392–446), EGF3 (450–489), cbEGF4 (490–529), cbEGF5 (530–571), cbEGF6 (572–612), cbEGF7 (613–653) and TB2 (656–717) corresponding to the domain boundaries. Domains were generated by homology modelling using Swiss-Model [44], or REMD for the proline-rich region. Domains were opened in UCSF Chimera [45] and combined using Modeller to model the missing linkers [46]. Rigid body modelling was completed through the BilboMD online portal [26] (https://bl1231.als.lbl.gov/bilbomd). The sample space was specified as *R_g_* of 30–70 Å (± 20 Å of the experimental *R_g_*) and run with 200 conformations per *R_g_* value to get appropriate sampling. Through the BilboMD pipeline, the models are passed to FoXS and MultiFoXS servers [47,48] to determine the ensemble of models that best represent the experimental SAXS data.

### Elastic network models and normal mode analysis

Elastic network models of TE, fibrillin-1 fragment PF2 and the TE–fibrillin complex (TE–PF2) were constructed by arranging beads along a regular lattice derived from the *ab initio* models. The elastic network models were constructed based on the DAMAVER averaged density, as in our previous work [29]. For consistency, for TE, the elastic network model was based on the previously published TE model [2], whereas for PF2 and the complex, the models shown in Figure 3, Figure 5 were used. Normal mode analysis of the elastic network model was conducted using the anisotropic network model in ProDy [49,50]. Network models were created by varying the cut-off distances of the interacting beads to capture nearest and second-nearest neighbours. Visualisation was performed in visual molecular dynamics [51]. RMSF of each structure was analysed with in-house scripts in visual molecular dynamics.

Normal mode analysis provides insight into the vibrational motion of a harmonic sinusoidal system in the small range about its equilibrium. This vibrational motion arises from a molecule's ability to sample an ensemble of conformations [49,52]. The conformational variations accessible to the model are confined to a range about a global energy minimum that defines the molecule's native state [49]. Normal modes are characterised by sinusoidal motion in which all parts of the system move with the same frequency and in phase. The normal modes of a system are independent from one another. Global modes of motion are the most cooperative motions, occurring at low frequency and engaging large subdomains within the structures. These low-frequency modes dominate the global motion of a molecule because the amplitude of oscillation for a mode is inversely proportional to the frequency of motion [29]. The potential is expressed as:

(1) $$V\left(u_{k}\right)=\frac{1}{2}u_{k}^{T}Hu_{k}=\frac{\omega_{k}^{2}}{2}$$

where *H* is the mass-weighted Hessian matrix, *u*_*k*_ is the set of solutions of the equations of motion and *⍵*_*k*_ are their frequencies of motion. The eigenvectors of the matrix form an orthonormal basis set, the normal modes of the molecule.

## CRediT authorship contribution statement

**Michael P. Lockhart-Cairns:** Investigation, Formal analysis, Writing - original draft. **Helena Newandee:** Formal analysis, Writing - original draft. **Jennifer Thomson:** Resources, Investigation. **Anthony S. Weiss:** Resources, Writing - original draft. **Clair Baldock:** Conceptualization, Project administration, Writing - review & editing. **Anna Tarakanova:** Methodology, Formal analysis, Writing - original draft.

## Appendix A Supplementary data

Supplementary material Image 1
