# Supplementary material for: Transglutaminase-Mediated Cross-Linking of Tropoelastin to Fibrillin Stabilises the Elastin Precursor Prior to Elastic Fibre Assembly
Source: J Mol Biol. 2020 Oct 2;432(21):5736–51. doi: 10.1016/j.jmb.2020.08.023 (PMC7610145; doi:10.1016/j.jmb.2020.08.023)
Supplement: Supplementary file 1 — Supplementary material [file mmc1.pdf]

Supplementary Figure 1

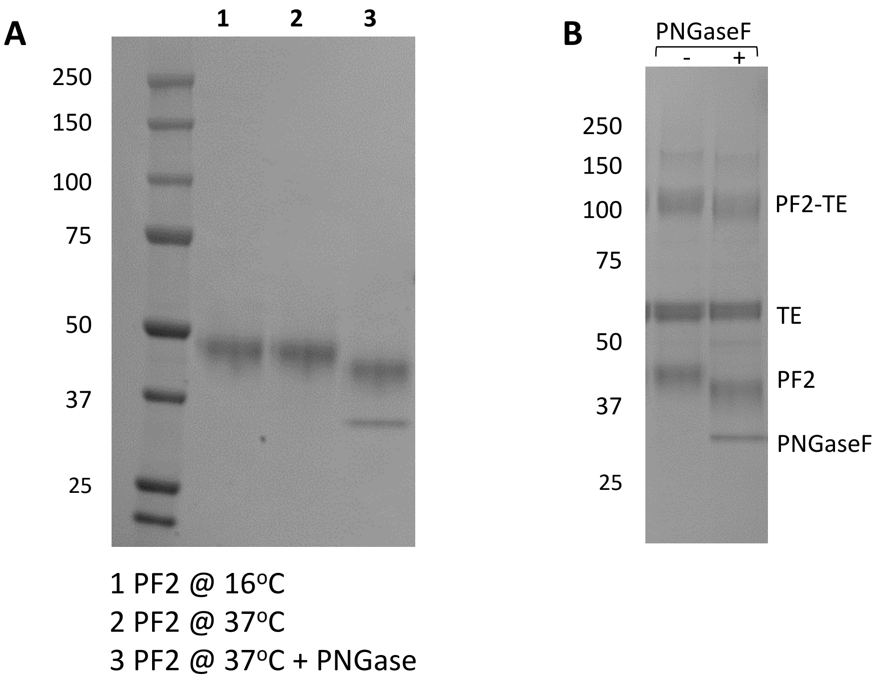

Supplementary Figure 1: Native deglycosylation of PF2 and PF2-TE complex still forms with deglycosylated PF2. (A) SDS-PAGE gel showing that PF2 can be deglycosylated in native conditions with PNGaseF. Native deglycosylation is performed at 37°C so PF2 was incubated at this temperature as a control to check for protein stability. (B) A PF2-TE complex still forms with natively deglycosylated PF2 indicating that N-glycosylation does not effect TG2 cross-link formation. Cross-linking was performed at 16°C.

Supplementary Figure 2

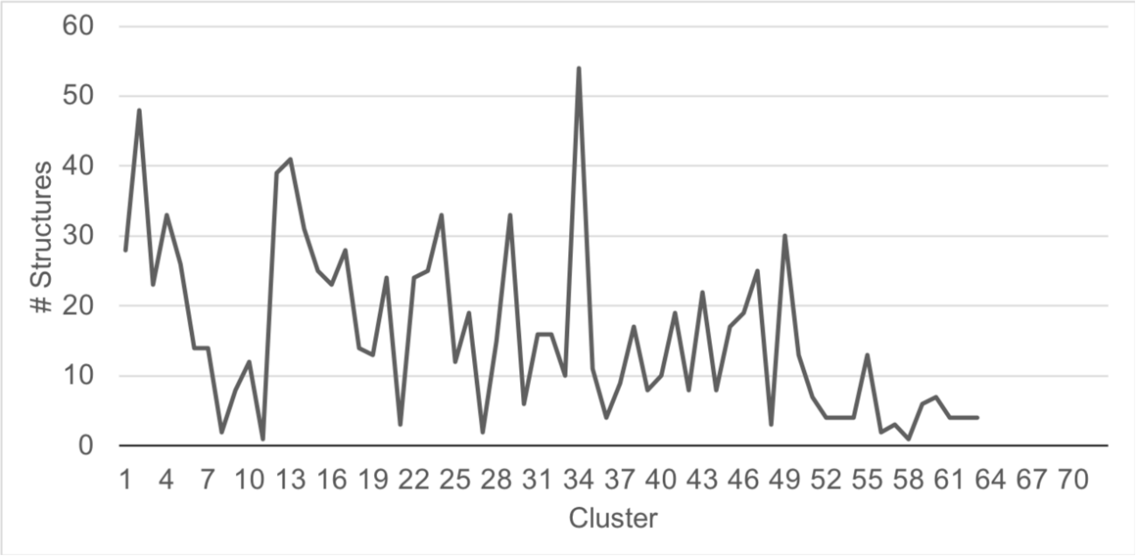

Supplementary Figure 2: The distribution of structures across clusters in the analysis of REMD simulations of the proline-rich region of PF2 suggests high structural flexibility and conformational range.

Supplementary Figure 3

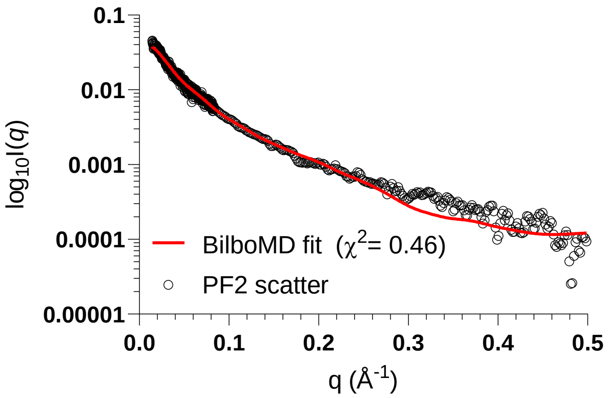

Supplementary Figure 3: Small angle X-ray scattering data of PF2 plotted in black as a function of resolution, with the fit to the BilboMD ensemble shown in red.

Supplementary Figure 4

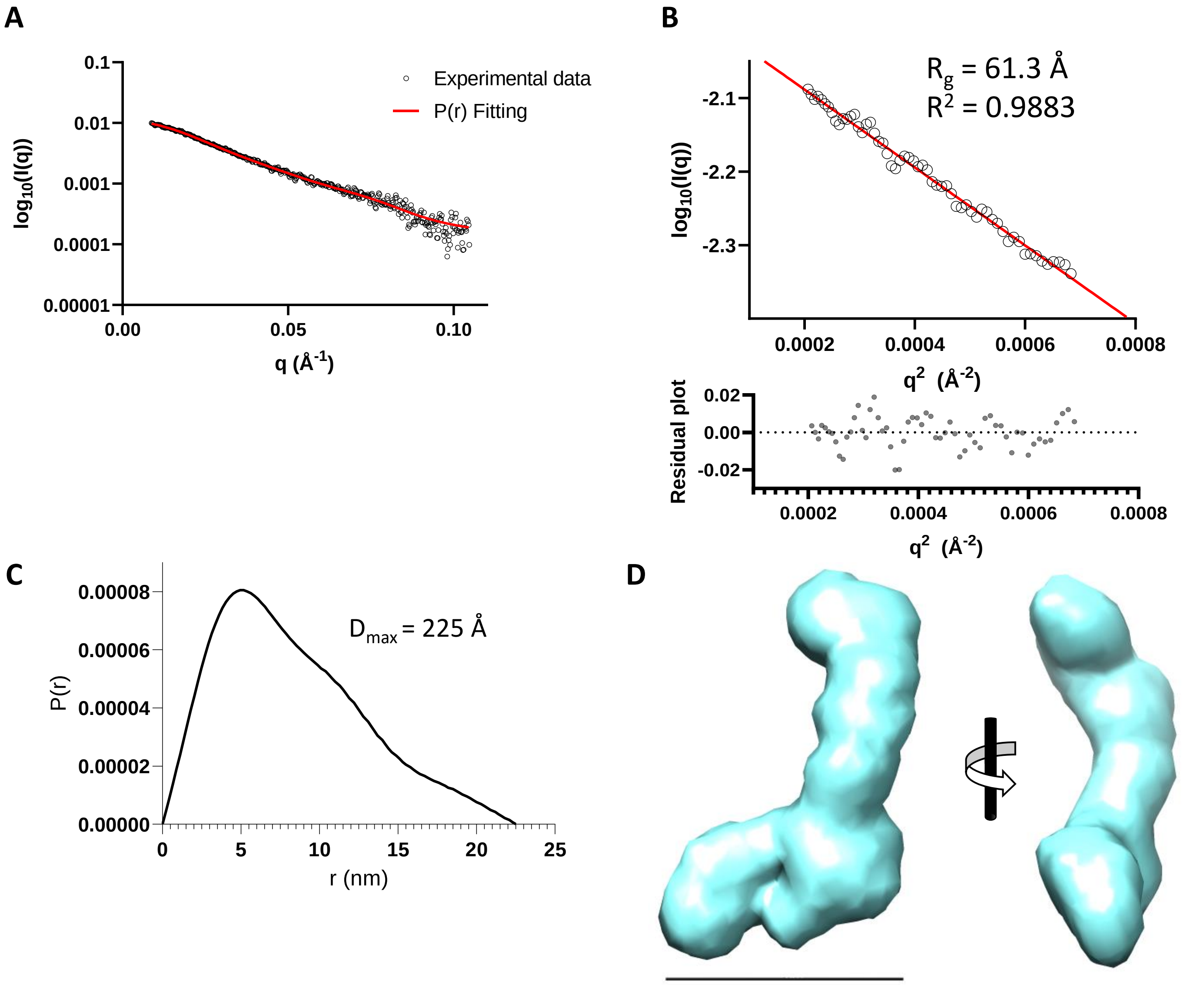

Supplementary Figure 4: (A) Small angle X-ray scattering data of TE plotted as a function of resolution. The indirect Fourier transform fitting to determine the pair-distance distribution function P(r) is shown in red. (B) The low q scattering data represented as a Guinier plot which is linear for values q ≤ 1/Rg. From the Guinier plot, the Rg can be estimated as 61.3 Å. (C) The pair-distance distribution function P(r) for TE shows a maximum dimension of 225 Å. (D) Using Gasbor, ab initio models of TE were calculated and the filtered average model is shown in two orthogonal orientations. Scale bar = 10 nm.

Supplementary Table 1

	PF2 (new)	PF2 (Ref: 22)	TE (Ref: 2)	TE (new)	Complex
**Protein Properties**					
Organism	*Homo sapiens*				
Uniprot sequence ID (residues)	P35555 (330-722 + LVPRGSHHHHHH)		P15502-2 (27-724) isoform 2		N/A
Mass (Da)	44211.35 (+ glycan)		60016.95		104228.30 (+ glycan)
Extinction coefficient [A_{280}, 0.1%(*w/v)]*	0.729		0.342		0.497
Protein concentration	1 mg/ml	1.8 mg/ml	5 mg/ml	10 mg/ml	1 mg/ml
**Data Collection Parameters**					
Source	Diamond Light Source	European Synchrotron Radiation Facility		Diamond Light Source	Diamond Light Source
Beamline	B21	ID02	ID02	B21	B21
Wavelength (Å)	0.99	1	1	0.99	0.99
Camera Length (mm)	4 m	1 m	1m and 5m	4 m	4 m
q measurement range (Å^{-1})	0.005 < q < 0.4	0.072 < q < 0.69	0.0038 < q < 0.53	0.005 < q < 0.4	0.005 < q < 0.4
Detector	Pilatus 2M	Thomson X-ray Intensifier (TH 49–427) lens coupled to a FReLoN CCD camera		Pilatus 2M	Pilatus 2M
Monitoring Radiation Damage	Frame by frame comparison				
Method of collection	Batch				
Exposure [frame × time(s)]	10 × 3	10 x 0.1		10 × 3	10 × 3
Buffer used	TBS	TBS + 5% glycerol	PBS	TBS	TBS

Supplementary Table 2

Software employed for SAXS data reduction, analysis and interpretation.	
SAXS data reduction	DAWN
Protein Parameter estimates	ProtParam
Basic Analysis	ScÅtter<br>Primus
Bead modelling	DAMMIF<br>GASBOR
Atomistic Modelling	BilboMD
Missing sequence modelling	Modeller
Graphical representation	UCSF Chimera – 1.14

Supplementary Table 3

	PF2	TE	Complex
Guinier Analysis			
I(0) (cm^{-1})	0.0414	0.012	0.0177
R_g (Å)	42.6	61.3	73.4
P(r) analysis			
I(0) (cm^{-1})	0.0414	0.011	0.164
R_g (Å)	45.7	66.2	71.0
D_{max} (Å)	150	225	234
q_{range} ($Å^{-1}$)	0.023 – 0.432	0.010 – 0.310	0.0085 – 0.2615
Mass estimation (kDa)			
MoW	41.96	54.5	154.96
V_c	42.79	55.0	162.33
Accession numbers (SASDBD)			
DAMMIF – defaults	0.036	N/A	1.10 – 1.14
GASBOR – 10 runs	N/A	1.3 – 2.4	N/A
BilboMD			
χ^2	0.47	N/A	N/A
